# A Single Center Analysis of Thymic Neuroendocrine Tumors †

**DOI:** 10.3390/cancers14194944

**Published:** 2022-10-09

**Authors:** Yirui Zhai, Qiang Zeng, Nan Bi, Zongmei Zhou, Zefen Xiao, Zhouguang Hui, Dongfu Chen, Luhua Wang, Jianyang Wang, Wenyang Liu, Lei Deng, Jima Lv, Wenqing Wang, Yang Luo, Junling Li, Xin Wang, Tao Zhang, Yushun Gao, Qinfu Feng

**Affiliations:** 1 Department of Radiation Oncology, National Cancer Center/National Clinical Research Center for Cancer/Cancer Hospital, Chinese Academy of Medical Sciences & Peking Union Medical College, Beijing 100021, China; 2 Department of VIP Medical Services, National Cancer Center/National Clinical Research Center for Cancer/Cancer Hospital, Chinese Academy of Medical Sciences & Peking Union Medical College, Beijing 100021, China; 3 Department of Radiation Oncology, National Cancer Center/National Clinical Research Center for Cancer/Cancer Hospital and Shenzhen Hospital, Chinese Academy of Medical Sciences & Peking Union Medical College, Shenzhen 518000, China; 4 Department of Medical Oncology, National Cancer Center/National Clinical Research Center for Cancer/Cancer Hospital, Chinese Academy of Medical Sciences & Peking Union Medical College, Beijing 100021, China; 5 Department of Thoracic Surgery, National Cancer Center/National Clinical Research Center for Cancer/Cancer Hospital, Chinese Academy of Medical Sciences & Peking Union Medical College, Beijing 100021, China

**Keywords:** thymic carcinomas, thymic neuroendocrine tumors, resection, radiotherapy, chemotherapy

## Abstract

**Simple Summary:**

Thymic neuroendocrine tumors are extremely rare and therefore few published studies currently exist. This study sought to investigate the basic clinical characteristics, treatment and prognosis of TNETs using single-center retrospective data and to address this gap in the literature. We found that thymic neuroendocrine tumors are a rare and aggressive disease with a high recurrence rate even in typical carcinoid tumors that are usually considered to have a good prognosis, with local recurrence and bone metastases being a common mode of treatment failure. Despite the widely accepted view that surgical resection should be the treatment of choice for thymic neuroendocrine tumors, we found that combination therapy including radiotherapy and chemotherapy is necessary to address the high recurrence rate of this typically aggressive tumor. In addition, patients who suffered from large vessel invasion in our study had a lower rate of overall survival and a high risk of tumor progression, other therapeutic regimes should be explored for these patients.

**Abstract:**

Purpose: Thymic neuroendocrine tumors (TNETs) are a collection of slow-progressing neoplasms located in the anterior mediastinum. Relatively few previously published studies have focused on thymic carcinomas. This study investigated the basic clinical characteristics, treatment, and prognosis of TNETs. Methods: Patients were enrolled in the study from January 2003 to December 2017 who had been diagnosed with TNETs through pathological screening and treated at our institution. Demographic data from each patient, the Masaoka stage, histology and size of the tumor, tumor invasion characteristics, and therapeutic strategies were gathered. The Kaplan–Meier method was used to assess patient survival. In addition, the log-rank test was used to carry out univariate analyses. Results: Twenty-six patients were eligible for inclusion in the study. The median age of the patients was 46.5 (25–69) years. The tumor median maximum diameter was 7.9 cm (from 3 to 19 cm). Twenty-four patients were treated surgically. Nineteen patients completed radiation therapy, and sixteen patients underwent chemotherapy. A median follow-up time of 54.95 months was observed. The survival rate for three years was 75.0% and 70.6% for five years. The corresponding progression-free survival rates for three and five years were 55.7% and 37.7%, respectively. The local, regional recurrence-free survival (LRFS) rates were 87.2% and 81.7%, and the distant metastasis-free survival (DMFS) rates were 55.7% and 37.7%, at three and five years, respectively. Local recurrence (six patients) and bone metastasis (six patients) were observed as the most frequent failures. Conclusion: TNET was observed to be an aggressive but rare malignant lesion. While the predominant treatment was complete resection, chemotherapy and radiotherapy were also required due to the high recurrence rate.

## 1. Introduction

Thymic epithelial neoplasms comprise a collection of rare neoplasms with a rate of morbidity at approximately 1.5 per one million people. It has been reported that thymic neuroendocrine tumors (TNETs) are even rarer, comprising less than 5% of all thymic tumors [1,2]. The incidence of TNETs was 0.1 per million people from 2001 to 2005 [3]. TNETs are distinct from thymomas, are composed of neuroendocrine cells, and exhibit more aggressive characteristics [4,5]. Currently, the incidence of TNETs is highly sporadic, limiting the ability to perform large clinical trials. Thus, the existing literature on TNET is limited. The purpose of this study was to provide additional evidence concerning the clinicopathological characteristics, proper therapeutic strategies, and prognostic factors of TNETs.

## 2. Materials and Methods

### 2.1. Ethics and Patient Enrollment

Ethical approval was waived by the local Ethics Committee of National Cancer Center/Cancer Hospital, Chinese Academy of Medical Sciences, and Peking Union Medical College in view of the retrospective nature of the study and because all of the procedures being performed were part of the routine care.

We assessed the medical reports from each patient treated at the National Cancer Center, Beijing, China, between January 2003 and December 2017. We also identified 26 patients in the thymic neoplasm database that were diagnosed with TNET based on a pathological assessment. 

### 2.2. Clinicopathological Characteristics

Information was collected for each patient, including demographics, tumor size, symptoms at initial presentation, smoking history, the status of invasiveness, histologic subtypes or grades, therapeutic protocols (resection, radiotherapy, and chemotherapy), and length of survival time. Before treatment, each patient received a physical examination as well as chest and abdominal computed tomography (CT). 

Tumor size was measured using pathologic diameters for patients who underwent surgery. For patients who did not undergo surgery, the tumor size was determined using CT. Two staging systems were used, including the American Joint Committee on Cancer TNM and the Masaoka systems. Typical carcinoid histology was classified as low-grade, while any atypical carcinoid histology was classified as an intermediate grade. Large cell neuroendocrine and small cell carcinomas were classified as high grade, according to the Fourth Edition of the World Health Organization Classification of Tumors.

### 2.3. Treatment

Individualized postoperative treatments for surgical patients were given by a multidisciplinary team based on the patient’s preoperative or postoperative tumor staging and pathological grading.

The surgical procedure was mainly carried out by complete removal of the tumor and the invaded tissue. Great vessel invasion is defined as tumor invasion of large vessels in the mediastinum, including the brachiocephalic vein, pulmonary vein, pulmonary artery, and aorta. In the patients with invasion of the brachiocephalic vein, the distal end of the brachiocephalic vein and the end of the confluent vena cava are blocked and the tumor and brachiocephalic vein are removed. For patients with invasion of large pulmonary vessels and the aorta, they may undergo gross total resection, partial resection, or biopsy.

### 2.4. Analysis of Outcomes

The status of the surgical margins was determined based on the intraoperative description of the surgical record and the microscopic presentation by the senior pathologist. Adverse events that occurred during treatment were assessed using documented data as proscribed by the National Cancer Institute Common Terminology Criteria for Adverse Events, Version 4.0. The responses of the tumors were evaluated by a senior radiologist and an oncologist by re-reviewing medical images, following the Response Evaluation Criteria in Solid Tumors (RECIST) Version 1.1. Recurrence and metastasis were obtained by medical records or telephone follow-up.

All survival rates were calculated starting from the time the diagnosis was made. The overall rate of survival (OS) was defined as the time from diagnosis to death, with death resulting from any cause. The time from diagnosis to a record of a documented clinical progression or death was defined as progression-free survival (PFS). The definition of local, regional relapse-free survival (LRFS) was determined as the time from diagnosis until a local, regional recurrence was noted. The distant metastasis-free survival (DMFS) was determined to be the time from diagnosis to the occurrence of a new distant metastasis. 

The Kaplan–Meier method was used to calculate the survival curves. The log-rank test was used to carry out the univariate analyses, and the following variables were included: Eastern Cooperative Oncology Group performance status score (ECOG PS); age; sex; paraneoplastic syndrome; smoking; Masaoka stage; TNM stage; histological grade; chemotherapy; radiotherapy; and R0 resection. A p-value less than 0.05 indicated statistical significance. SPSS Version 26.0 software (IBM Corp, Armonk, NY, USA) and R version 3.5.1 were used to conduct the statistical analyses.

## 3. Results

### 3.1. Patient Characteristics

The analysis included 26 patients. A median age of 46.5 (25–69) years was observed. The tumors exhibited a median maximum diameter of 7.9 cm (from 3 to 19 cm). Eight patients (30.8%) were asymptomatic. Chest pain (19.2%) and chest tightness (15.4%) were the most common symptoms. Superior vena cava suppression syndrome was observed in one patient. Paraneoplastic syndrome was observed in 3 of the 26 patients. In addition, Cushing’s syndrome, multiple endocrine neoplasia type 1, and myasthenia gravis were each recorded in one patient. The details of these cases are shown in Table 1. 

### 3.2. Treatments

Twenty-four patients underwent surgical resection. Nineteen patients completed radiotherapy, and sixteen patients completed chemotherapy. Six surgical patients (23.1%) had positive surgical margins, two of them underwent biopsy due to extensive involvement of surrounding tissues, two with large vessel invasion, one invading the pericardium, and one with bilateral vagal nerve invasion; three cases were treated with postoperative radiotherapy, two cases were treated with postoperative chemotherapy, and one case was treated with both postoperative radiotherapy and chemotherapy. The median dose of radiotherapy was 60 Gy (from 22 to 65 Gy), using three-dimensional radiation or intensity-modulated radiotherapy. Chemotherapy was determined to have a median cycle number of four (range of one to ten cycles). Eleven of the patients (68.8%) treated with chemotherapy were treated with a platinum-based chemotherapy regimen, including four with cyclophosphamide, doxorubicin, and cisplatin, and five with a regimen that included cisplatin and etoposide. Of the six patients with great vessel invasion, one did not undergo surgery, one underwent biopsy only, and one of the remaining four was treated with neoadjuvant chemotherapy. Twenty patients (76.9%) developed recurrence or metastasis, and after recurrence, ten patients received chemotherapy, four patients received radiotherapy, and two patients received multi-targeted tyrosine kinase inhibitors. The individual patients’ details and treatment strategies are shown in Table 2 and Table S1 (Supplementary Materials).

### 3.3. Survival

**Figure 1:**
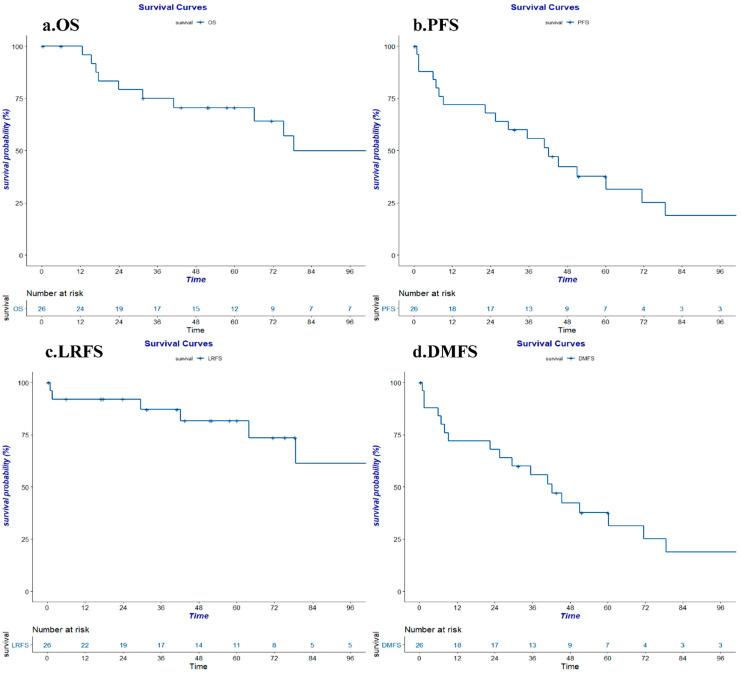
(**a**) OS: overall survival; (**b**) PFS: progression-free survival; (**c**) LRFS: local-regional relapse free survival; (**d**) DMFS: distant metastasis-free survival.

The median follow-up time was determined to be 54.95 months. Twelve patients had died by the time of the last follow-up. Six patients exhibited local tumor recurrence, while nineteen patients exhibited distant metastasis. None of the patients experienced only local recurrence. The three-year survival rate was 75.0%, and the five-year survival rate was 70.6%. Corresponding PFS rates were determined to be 55.7% and 37.7%, respectively. The three- and five-year LRFS were 87.2% and 81.7%, and the three- and five-year DMFS were 55.7% and 37.7%, respectively. The three- and five-year rate for low grade TNETs were 87.5% and 81.2%, for intermediate and for high grade TNETs were both 50.0% (see Supplementary Figure S1). The most commonly observed failure was local recurrence (six patients) and bone metastasis (six patients), followed by lung metastasis (five patients), pleural metastasis (five patients), and pancreatic metastasis (five patients). The calculated survival curves are shown in Figure 1a–d.

### 3.4. Toxicity

Toxicities of grade 3 or higher were exhibited by four patients. Three had leucopenia, and one presented with liver injury. All toxicities of grade 3 or higher were induced by chemotherapy and were resolved after medical intervention.

### 3.5. Prognostic Factors 

**Figure 2:**
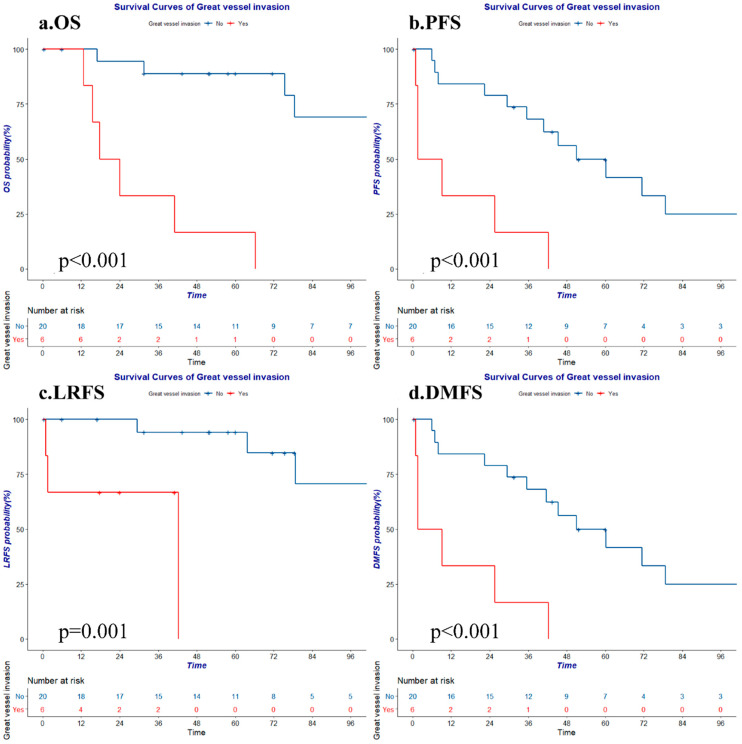
The OS, PFS, LRFS, and DMFS of lesions with or without great vessel invasion. (**a**) OS: overall survival; (**b**) PFS: progression-free survival; (**c**) LRFS: local-regional relapse free survival; (**d**) DMFS: distant metastasis-free survival.

**Figure 3:**
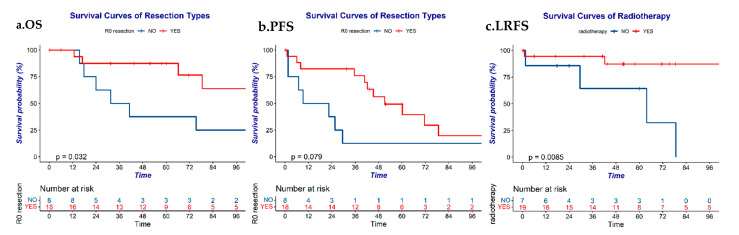
(**a**,**b**) The OS and PFS of lesions with or without complete resection. (**c**) The LRFS of lesions with or without radiotherapy. Abbreviation: OS: overall survival; PFS: progression-free survival; LRFS: local-regional relapse free survival.

Univariate analyses revealed that sex, the tumor histological grade, age, smoking, paraneoplastic syndrome, and chemotherapy all showed no significant association with survival. Major vessel invasion was a strong negative predictive factor for all four survival metrics (Figure 2). R0 resection was associated with favorable OS (Figure 3a). Because all patients with progression exhibited distant metastasis, the PFS results were the same as the DMFS results. Patients with an earlier Masaoka stage or who underwent R0 resection (Figure 3b) presented a relatively favorable PFS/DMFS, despite the fact that neither variable was closely associated with PFS or DMFS. Similarly, patients with an earlier Masaoka stage or who received radiotherapy exhibited a better LRFS than patients who did not (Figure 3c). The detailed analysis is shown in Table 3.

## 4. Discussion

TNET rarely occurs. Previous reports regarding this type of thymic malignancy have been primarily case reports and a few studies. In historical studies, males have been more likely to exhibit this disease than females (ratio: 2:1 to 5:1) [3,6,7,8,9,10,11,12,13,14,15,16,17]. The median age at diagnosis ranges from 46 to 61 years [1,3,6,7,8,9,10,11,12,13,14,15,16,17,18]. The gender and age patterns observed in our study were comparable to those described in previous studies. 

Paraneoplastic syndrome is a common characteristic of neuroendocrine neoplasms. The reported incidence of paraneoplastic syndrome in previous studies ranged from 7 to 36% [7,8,13,16,18]. The rate of paraneoplastic syndrome noted in this study was observed to be in the same range. As an invasive disease, TNET induces symptoms as adjacent organs are invaded. Chest pain was the most commonly reported symptom in two previous studies [9,14]. Similarly, the findings of our study were consistent with these previous reports. High invasion rates also result in advanced stages of the disease. Most patients exhibit disease at Masaoka stages III–IV at the time of diagnosis, as seen in several previous studies (from 55.1% to 75.8%) [6,8,11,15,16,17] and this study (61.5%).

Nevertheless, the OS of TNET was not disappointing. The five-year OS reported by previous studies ranged from 23.4% to 100% [6,7,8,9,11,12,13,14,15,16,17,18]. Similarly, the results observed in this study were within this same range. The wide range of OS in the historical reports could be due to differences in patient characteristics and treatments or the small number of patients included in many of the studies. To date, two large studies based on data obtained from the International Thymic Malignancy Interest Group as well as the European Society of Thoracic Surgeons databases, and the Surveillance, Epidemiology and End Results (SEER) database have revealed five-year OS rates of 68% and 56%, respectively [10,12]. Ose et al. reported a five-year PFS of 48% [14]. The corresponding data obtained from our study were not similar with respect to the PFS. A higher OS and lower PFS indicated a high rate of recurrence. Araki et al. reported that in 79% of patients, relapse was observed, including local recurrence in 50% of the patients and metastasis in 57.1% of the patients [13]. Similarly, Hamaji et al. reported a recurrence rate of 57.1%, including local recurrence in 28.6% of the patients and distant metastasis in 28.6% of the patients [17]. This pattern was consistent with the findings from our study. 

Previous studies have reported that bone and lung were the most prevalent sites of metastasis [7,14], and our study supported these results. Moreover, our study also observed a high rate of pleural and pancreatic metastases. These two metastatic sites could be related to the primary tumor site as well as the specific tumor histology. Pleural metastasis is a typical recurrence mode in thymic neoplasms [19]; meanwhile, the pancreas is a site of predilection for neuroendocrine neoplasms [20].

It is still controversial whether histological grades affect cancer prognosis. Theoretically, tumors with a higher histological grade are more invasive. Ma et al. reported that the histological grade is a prognostic factor for OS (*p* = 0.02) [11]. However, two other studies reported the opposite conclusion [7,16]. In addition, Corsini et al. reported that PFS was not affected by the tumor histological grade [16]. Although the findings of two large studies did not agree, both the multivariate analysis by Sullivan et al. and the univariate analysis by Filosso et al. suggested that histological grade is not a good prognostic factor for OS (*p* = 0.19 to 0.942) [10,12]. Combined with similar results from our study, we concluded that histologic subtype does not have a significant independent effect on survival for TNET.

The Masaoka staging system is currently the most widely used worldwide, but it has been shown to be substantially inaccurate in cases of TNET [7]. Our findings revealed that Masaoka stage I–II patients achieved superior OS, PFS, and DMFS compared with stage III–IV patients. The controversies described in previous reports could be related to limited sample sizes, as the number of patients included in most previous studies was between 14 to 40 patients. In addition, the Masaoka stage was observed to be an OS prognostic factor in several previously published large studies (*p* = 0.001 to 0.02) [10,12,21]. Thus, we suggest that the OS for patients with disease characterized by an advanced Masaoka stage was worse than patients presenting an earlier Masaoka stage. 

The American Joint Committee on Cancer and the International Thymic Malignancy Interest Group jointly explored a staging system for TNM in 2014 [22]. However, the validity of this new staging system for TNET is unknown as few study has investigated this topic as yet [23]. Thus, we evaluated the effectiveness of this new staging system in our study and determined that differences existed in OS, PFS, and DMFS between stages I–II and III–IV, although the *p*-value was higher than that observed for the Masaoka stages (*p* = 0.124 to 0.136 vs. *p* = 0.056 to 0.079). Therefore, which staging system is most appropriate for TNET should be investigated in future large studies. 

A few studies have suggested that the invasion of major vessels, including the aorta, pulmonary artery, superior vena vein, or brachiocephalic vein, substantially increases the challenges associated with successful R0 resection, causes more intraoperative and postoperative complications, and results in frequent metastasis in thymic neoplasms [24]. In our study, invasion of major vessels was a strong negative prognostic indicator for all survival rates (*p*=0.000 to 0.001). Patients with major vessel invasion exhibited an exceptionally poor prognosis, probably due to the lack of opportunities for surgical treatment. 

Surgery is the predominant treatment for TNET. Several studies have demonstrated that surgical resection (*p* = 0.001) was closely associated with increased survival [7,10,11,15,21]. R0 resection was the decisive factor that impacted survival in a series of studies [25]. Corsini et al. enrolled 49 patients with TNET and found that OS in patients who underwent R0 resection was improved compared with patients without surgical intervention (73% vs. 41%, *p* = 0.002) [16]. Similarly, Ose et al. reported a difference in OS between patients with and without R0 resection (*p* = 0.010) [14]. Finally, Filosso et al. indicated in their study that the only independent predictor for OS was R0 resection (*p* = 0.048) [10]. The results of our study also demonstrated the significance of surgery, especially R0 resection, in patients with TNET. R0 resection improved OS and might have improved PFS and DMFS. However, surgery did not influence LRFS. One possible reason is that patients who did not undergo R0 resection were more likely to receive radiotherapy when compared with patients who did undergo R0 resection.

A definite conclusion could not be made whether radiotherapy and chemotherapy helped improve survival [21,26,27,28,29], although it is generally accepted that TNET are more aggressive than thymomas, and adjuvant radiotherapy is now mostly recommended for aggressive thymomas. Corsini et al. found that neither radiation nor chemotherapy impacted OS (*p* = 0.594, *p* = 0.234) [16]. Our study supported these findings. However, Ose et al. found that ancillary radiotherapy and chemotherapy improved OS (*p* = 0.042) [14]. We only observed in our study an association between radiotherapy and LRFS. Nevertheless, a favorable LRFS in patients who underwent radiotherapy was not associated with an improved OS due to the occurrence of frequent distant metastases. Thus, chemotherapy was necessary, although patients who received chemotherapy did not exhibit an improved DMFS in our study. A possible reason could be the presence of a higher proportion of stage IV patients in the group that received chemotherapy. Based on a high proliferative activity and high sensitivity to chemotherapy observed with neuroendocrine tumors in historical studies, as well as a high DMFS, we believe that chemotherapy is necessary for the treatment of this disease.

In recent years, peptide receptor radionuclide therapy (PRRT) has evolved as an important modality in the treatment of advanced, metastatic or inoperable, progressive neuroendocrine neoplasms, with favorable tumor control results [30,31]. However, due to the rarity of TNET and the limitations of diagnostic and therapeutic options in the early years, PRRP is hardly accessible, and only a few patients were treated with PRRP in the back line, and one of them achieved longer tumor control. More data are needed to support the role of PRRP in TNET, but the important role of classical antitumor approaches in treatment should not be overlooked.

Wen et al. compared the characteristics of TNET and thymic carcinoma in 2018 based on the SEER database. In that study, more male patients presented with TNET (70% vs. 60%, *p* < 0.001), the patients were younger (59 years vs. 62 years, *p* < 0.001), there were fewer stage III–IV patients (29.5% vs. 34.8%, *p* < 0.001), and there were more cases of metastasis to lymph nodes (57% vs. 33%, *p* < 0.001) [15]. The results of Wen et al. indicated that the OS for TNET was better than thymic carcinoma. Our results also show a relatively modest OS.

There were multiple advantages associated with our study. First, we conducted a single-center study that focused on an exceedingly rare disease and provided additional evidence concerning TNET. Second, all patients enrolled in the last 20 years were treated in the same medical center. These patients received modern treatment technologies, including video-assisted thoracic surgery and intensity-modulated radiotherapy that represented the current treatment status. Third, we evaluated the accuracy of the two main staging systems, Masaoka, and TNM staging. To our knowledge, this is the first study to use a new system to evaluate the prognosis of TNET. 

In addition, some limitations were noted in our study. First, we utilized coded administrative data to identify toxicities associated with treatment. This procedure might have produced a less comprehensive analysis when compared with prospective trials. Second, our retrospective analysis spans, as expected for such a rare disease, over a long period and have a quite heterogeneous population in combination with a heterogeneous therapeutic approach. Third, surgical complications were not reported in this study and are difficult to track at this time due to the long duration of treatment in a retrospective study. Besides, the overall sample size was still small due to the rarity of the disease.

## 5. Conclusions

TNET is an aggressive but rare malignant disease that exhibits a number of characteristics associated with neuroendocrine tumors as well as a high recurrence rate. Although the predominant treatment has been determined to be complete resection, it is also the case that the application of radiotherapy and chemotherapy is necessary. On the other hand, patients with great vessel invasion exhibited worse survival rates. Therefore, other therapeutic regimes should be explored for these patients. 

## Figures and Tables

**Table 1:** Patient Characteristics.

		Number of Patients *n* = 26	%
Age	Median (range)	45 (25–69)	
Sex	Male	20	76.9
	Female	6	23.1
Histological grade	Low	17	65.4
	Intermediate	6	23.1
	High	3	11.5
ECOG PS score	0–1	23	88.5
	≥2	3	11.5
Smoking	Yes	12	46.2
	No	14	53.8
Paraneoplastic syndrome	Yes	3	11.5
	No	23	88.5
Great vessel invasion	Yes	6	23.1
	No	20	76.9
Masaoka stage	I	2	7.7
	II	8	30.8
	III	9	34.6
	IVa	1	3.8
	IVb	6	23.1
T stage	T1	10	38.5
	T2	1	3.8
	T3	12	46.2
	T4	3	11.5
N stage	N0	24	92.3
	N1	2	7.7
M stage	M0	20	76.9
	M1a	1	3.8
	M1b	5	19.2
TNM stage	I	10	38.5
	II	1	3.8
	IIIa	7	26.9
	IIIb	1	3.8
	IVa	2	7.7
	IVb	5	19.2

**Table 2:** Treatment Strategies.

Treatment		Number of Patients	%
Surgery	R0	18	69.2
	R1/R2	6	23.1
	No	2	7.7
Radiation	Yes	19	73.1
	No	7	26.9
Chemotherapy	Yes	16	61.5
	No	10	38.5
Therapeutic regimens	Surgery alone	1	3.8
	Surgery + radiotherapy	9	34.6
	Surgery + chemotherapy	4	15.4
	Surgery + chemoradiotherapy	9	34.6
	Chemoradiotherapy + surgery	1	3.8
	Chemotherapy alone	2	7.7

**Table 3:** Univariate analysis of survival.

		5y OS		5y PFS		5y LRFS		5y DMFS	
		%	*p*	%	*p*	%	*p*	%	*p*
Sex	Male	73.3	0.387	42.7	0.451	87.7	0.589	42.7	0.451
	Female	60.0		20.0		60.0		20.0	
Histological grade	Low	81.3	0.293	35.7	0.359	86.5	0.461	35.7	0.359
	Medium	60.0		50.0		83.3		50.0	
	High	33.3		33.3		50.0		33.3	
Age	≤60y	68.0	0.637	43.6	0.455	82.5	0.551	43.6	0.455
	>60y	80.0		20.0		80.0		20.0	
ECOG PS score	≤1	71.4	0.278	40.0	0.817	90.9	**0.016**	40.0	0.817
	≥2	66.7		0.0		0.0		0.0	
Smoking	Yes	62.3	0.836	42.4	0.753	75.8	0.814	42.4	0.753
	No	76.9		34.3		84.4		34.3	
Masaoka stage	I–II	90.0	0.079	67.5	0.056	100.0	0.516	67.5	0.056
	III–IV	57.1		20.0		68.3		20.0	
TNM stage	I–II	90.9	0.136	61.4	0.124	100.0	0.372	61.4	0.124
	III–IV	53.8		21.4		65.3		21.4	
Great vessel invasion	Yes	16.7	**0.000**	0.0	**0.000**	0.0	**0.001**	0.0	**0.000**
	No	88.9		49.9		94.1		49.9	
R0 resection	Yes	87.5	**0.032**	49.3	0.079	86.9	0.812	49.3	0.079
	No	37.5		12.5		70.0		12.5	
Radiation	Yes	81.9	0.533	34.6	0.468	87.2	**0.009**	34.6	0.468
	No	42.9		42.9		64.3		42.9	
Chemotherapy	Yes	56.3	0.366	45.7	0.913	66.9	0.137	45.7	0.913
	No	90.0		30.0		100.0		30.0	
Paraneoplastic syndrome	Yes	100.0	0.409	35.4	0.612	100.0	0.496	35.4	0.612
	No	68.2		66.7		100.0		66.7	

ECOG PS: Eastern Cooperative Oncology Group performance status; LRFS: local, regional relapse-free survival; DMFS: distant, metastasis-free survival; PFS: progression-free survival; OS: overall survival.

## Data Availability

The data presented in this study are available in the article.

## Supplementary Materials

The following supporting information can be downloaded at: https://www.mdpi.com/article/10.3390/cancers14194944/s1, Figure S1: The overall survival curve of low versus intermediate/high grade TNET; Table S1: Individual patient data about clinicopathological characteristics, treatment information and outcomes.

## References

[1] de Jong W.K., Blaauwgeers J.L., Schaapveld M., Timens W., Klinkenberg T.J., Groen H.J. (2008). Thymic epithelial tumours: A population-based study of the incidence, diagnostic procedures and therapy. Eur. J. Cancer.

[2] Phan A.T., Oberg K., Choi J., Harrison L.H., Hassan M.M., Strosberg J.R., Krenning E.P., Kocha W., Woltering E.A., Maples W.J. (2010). NANETS consensus guideline for the diagnosis and management of neuroendocrine tumors: Well-differentiated neuroendocrine tumors of the thorax (includes lung and thymus). Pancreas.

[3] Hsu C.H., Chan J.K., Yin C.H., Lee C.C., Chern C.U., Liao C.I. (2019). Trends in the incidence of thymoma, thymic carcinoma, and thymic neuroendocrine tumor in the United States. PLoS ONE.

[4] Oberg K., Jelic S., Group E.G.W. (2009). Neuroendocrine bronchial and thymic tumors: ESMO clinical recommendation for diagnosis, treatment and follow-up. Ann. Oncol..

[5] Volante M., Mete O., Pelosi G., Roden A.C., Speel E.J.M., Uccella S. (2021). Molecular pathology of well-differentiated pulmonary and thymic neuroendocrine tumors: What do pathologists need to know?. Endocr. Pathol..

[6] Fukai I., Masaoka A., Fujii Y., Yamakawa Y., Yokoyama T., Murase T., Eimoto T. (1999). Thymic neuroendocrine tumor (thymic carcinoid): A clinicopathologic study in 15 patients. Ann. Thorac. Surg..

[7] Crona J., Bjorklund P., Welin S., Kozlovacki G., Oberg K., Granberg D. (2013). Treatment, prognostic markers and survival in thymic neuroendocrine tumours. A study from a single tertiary referral centre. Lung Cancer.

[8] Strobel P., Zettl A., Shilo K., Chuang W.Y., Nicholson A.G., Matsuno Y., Gal A., Laeng R.H., Engel P., Capella C. (2014). Tumor genetics and survival of thymic neuroendocrine neoplasms: A multi-institutional clinicopathologic study. Genes Chromosomes Cancer.

[9] Weissferdt A., Kalhor N., Liu H., Rodriguez J., Fujimoto J., Tang X., Wistuba I.I., Moran C.A. (2014). Thymic neuroendocrine tumors (paraganglioma and carcinoid tumors): A comparative immunohistochemical study of 46 cases. Hum. Pathol..

[10] Filosso P.L., Yao X., Ahmad U., Zhan Y., Huang J., Ruffini E., Travis W., Lucchi M., Rimner A., Antonicelli A. (2015). Outcome of primary neuroendocrine tumors of the thymus: A joint analysis of the International Thymic Malignancy Interest Group and the European Society of Thoracic Surgeons databases. J. Thorac. Cardiovasc. Surg..

[11] Ma K., Liu Y., Xue Z., Chu X. (2017). Treatment, prognostic markers, and survival in thymic neuroendocrine tumors: A single center experience of 41 patients. Medicine.

[12] Sullivan J.L., Weksler B. (2017). Neuroendocrine tumors of the thymus: Analysis of factors affecting survival in 254 patients. Ann. Thorac. Surg..

[13] Araki T., Sholl L.M., Hatabu H., Nishino M. (2018). Radiological features and metastatic patterns of thymic neuroendocrine tumours. Clin. Radiol..

[14] Ose N., Maeda H., Inoue M., Morii E., Shintani Y., Matsui H., Tada H., Tokunaga T., Kimura K., Sakamaki Y. (2018). Results of treatment for thymic neuroendocrine tumours: Multicentre clinicopathological study. Interact. Cardiovasc. Thorac. Surg..

[15] Wen J., Chen J., Chen D., Liu D., Xu X., Huang L., Cao J., Zhang J., Gu Y., Fan M. (2018). Evaluation of the prognostic value of surgery and postoperative radiotherapy for patients with thymic neuroendocrine tumors: A propensity-matched study based on the SEER database. Thorac. Cancer.

[16] Corsini E.M., Mitchell K.G., Sceusi E.L., Mehran R.J., Rice D.C., Sepesi B., Walsh G.L., Swisher S.G., Roth J.A., Vaporciyan A.A. (2019). Multidisciplinary treatment of thymic neuroendocrine tumors: Surgery remains a key component. J. Thorac. Dis..

[17] Hamaji M., Omasa M., Nakagawa T., Miyahara S., Suga M., Kawakami K., Aoyama A., Date H. (2020). Survival outcomes of patients with high-grade and poorly differentiated thymic neuroendocrine carcinoma. Interact. Cardiovasc. Thorac. Surg..

[18] de Montpreville V.T., Macchiarini P., Dulmet E. (1996). Thymic neuroendocrine carcinoma (carcinoid): A clinicopathologic study of fourteen cases. J. Thorac. Cardiovasc. Surg..

[19] Zhai Y., Hui Z., Ji W., Wang X., Liang J., Mao Y., Luo Y., Zou S., Lv J., Zhou Z. (2017). A single-center analysis of the treatment and prognosis of patients with thymic carcinoma. Ann. Thorac. Surg..

[20] Taskin O.C., Clarke C.N., Erkan M., Tsai S., Evans D.B., Adsay V. (2020). Pancreatic neuroendocrine neoplasms: Current state and ongoing controversies on terminology, classification and prognostication. J. Gastrointest. Oncol..

[21] Chen Y., Zhang J., Zhou M., Guo C., Li S. (2022). Real-world clinicopathological features and outcome of thymic neuroendocrine tumors: A retrospective single-institution analysis. Orphanet. J. Rare Dis..

[22] Carter B.W., Benveniste M.F., Madan R., Godoy M.C., Groot P.M., Truong M.T., Rosado-de-Christenson M.L., Marom E.M. (2017). IASLC/ITMIG staging system and lymph node map for thymic epithelial neoplasms. Radiographics.

[23] Fang W., Filosso P.L., Roden A.C., Gu Z., Liu Y., Agzarian J., Shen R.K., Ruffini E. (2021). Clinicopathological features and current treatment outcomes of neuroendocrine thymic tumours. Eur. J. Cardiothorac. Surg..

[24] Comacchio G.M., Dell’Amore A., Marino M.C., Russo M.D., Schiavon M., Mammana M., Faccioli E., Lorenzoni G., Gregori D., Pasello G. (2021). Vascular involvement in thymic epithelial tumors: Surgical and oncological outcomes. Cancers.

[25] Filosso P.L., Yao X., Ruffini E., Ahmad U., Antonicelli A., Huang J., Guerrera F., Venuta F., van Raemdonck D., Travis W. (2016). Comparison of outcomes between neuroendocrine thymic tumours and other subtypes of thymic carcinomas: A joint analysis of the European Society of Thoracic Surgeons and the International Thymic Malignancy Interest Group. Eur. J. Cardiothorac. Surg..

[26] Kundel Y., Yellin A., Popovtzer A., Pfeffer R., Symon Z., Simansky D.A., Oberman B., Sadezki S., Brenner B., Catane R. (2007). Adjuvant radiotherapy for thymic epithelial tumor: Treatment results and prognostic factors. Am. J. Clin. Oncol..

[27] Gaur P., Leary C., Yao J.C. (2010). Thymic neuroendocrine tumors: A SEER database analysis of 160 patients. Ann. Surg..

[28] Jia R., Sulentic P., Xu J.M., Grossman A.B. (2017). Thymic neuroendocrine neoplasms: Biological behaviour and therapy. Neuroendocrinology.

[29] Bian D., Qi M., Hu J., Ning Y., Zhou F., Fei K., Zhang P. (2018). The comparison of predictive factors regarding prognoses and invasion of thymic neuroendocrine tumors preoperatively and postoperatively. J. Thorac. Dis..

[30] Baum R.P., Kulkarni H.R., Singh A., Kaemmerer D., Mueller D., Prasad V., Hommann M., Robiller F.C., Niepsch K., Franz H. (2018). Results and adverse events of personalized peptide receptor radionuclide therapy with (90)Yttrium and (177)Lutetium in 1048 patients with neuroendocrine neoplasms. Oncotarget.

[31] Ambrosini V., Kunikowska J., Baudin E., Bodei L., Bouvier C., Capdevila J., Cremonesi M., de Herder W.W., Dromain C., Falconi M. (2021). Consensus on molecular imaging and theranostics in neuroendocrine neoplasms. Eur. J. Cancer.

