# Supplementary material for: A Single Center Analysis of Thymic Neuroendocrine Tumors
Source: Cancers (Basel). 2022 Oct 9;14(19):4944. doi: 10.3390/cancers14194944 (PMC9564064; doi:10.3390/cancers14194944)

Table S1. Individual patient data about clinicopathological characteristics、treatment information and outcomes.

ID	Sex	Age	ECOG	Smoking	histologic grades	Masaoka stage	Tumor Size(cm)	Paraneoplastic syndrome	Treatment	Resection margin	RT dose (Gy)	Chemotherapy cycles
**1**	male	63	1	Yes	G1	III	9.0	No	S+R	R0	50	/
**2**	female	61	1	No	G1	IVb(N+)	7.0	No	S+R	R0	60	/
**3**	male	59	1	No	G1	II	5.0	No	S+R	R0	60	/
**4**	male	25	1	No	G1	I	8.0	No	S+R	R0	60	/
**5**	female	67	1	No	G1	III	5.0	No	S+R	R1/R2	65	/
**6**	male	54	1	Yes	G1	II	3.0	No	S+R	R0	60	/
**7**	male	60	1	No	G1	III	14.0	No	S+R	R1/R2	52	/
**8**	male	38	1	No	G2	III	5.0	No	S+R	R1/R2	60	/
**9**	male	34	1	No	G1	III	3.0	Yes	S+R	R0	50	/
**10**	female	42	1	Yes	G2	II	10.0	No	S+R+C	R0	54	4
**11**	male	44	1	Yes	G1	IVa	9.0	No	S+R+C	R0	50	4
**12**	male	47	2	Yes	G1	III	8.2	No	S+R+C	R0	60	4
**13**	male	32	1	Yes	G1	III	12.0	No	S+R+C	R1/R2	60	5
**14**	male	40	2	No	G2	III	NA	Yes	S+R+C	R0	50	4
**15**	male	45	1	Yes	G2	IVb(N+)	15.0	No	S+R+C	R0	60	4
**16**	male	46	1	Yes	G2	II	7.0	No	S+R+C	R0	60	4
**17**	male	32	1	No	G1	II	6.0	No	S+R+C	R0	50	4
**18**	male	51	2	Yes	G3	II	3.0	Yes	S+R+C	R0	50	4
**19**	male	66	1	No	G1	II	11.0	No	S	R0	/	/
**20**	male	30	1	Yes	G1	III	19.0	No	C+R+S	R0	22	1
**21**	male	34	1	Yes	G1	I	5.0	No	S+C	R0	/	4
**22**	female	37	1	No	G1	IVb	10.0	No	S+C	R1/R2	/	8
**23**	male	58	1	Yes	G3	IVb	8.0	No	S+C	R1/R2	/	6
**24**	male	48	1	No	G1	II	4.0	No	S+C	R0	/	10

**25**	female	69	2	No	G3	IVb	NA	No	C	No surgery	/	8
**26**	male	44	2	No	G2	IVb	6.8	No	C	No surgery	/	5

Continued.

**ID**	**Chemotherapy regimens**	**Survival status**	**PFS months**	**OS months**	**Recurrence sites**	**Treatment regimens after recurrence**
**1**	/	alive	51.09	52.11	pancreas、bone	no treatment
**2**	/	alive	45.31	71.59	cervical lymph node	no treatment
**3**	/	alive	40.84	57.79	pancreas、bone	C
**4**	/	die	35.52	78.52	pancreas、abdominal lymph node	R
**5**	/	die	22.44	102.54	lung	no treatment
**6**	/	die	7.95	16.99	bone	R
**7**	/	die	6.93	75.37	lung	C
**8**	/	alive	155.14	155.14	/	/
**9**	/	alive	51.65	51.65	/	/
**10**	cyclophosphamide + doxorubicin + cisplatin	die	104.84	145.91	pleural、pancreas、	C
**11**	cyclophosphamide + doxorubicin + cisplatin	alive	71.49	120.41	SLN, pleura, pancreas、bone	C
**12**	non-platinum regimen	die	42.25	66.27	mediastinum, pleura, adrenal, liver	C
**13**	Paclitaxel + cisplatin + etoposide	die	25.59	41.10	liver, pleura, adrenal, pancreas,	C + multiple TKIs
**14**	non-platinum regimen	alive	6.00	6.04	kidney	S
**15**	vinorelbine + cisplatin	die	0.95	12.71	mediastinum, lung	C
**16**	cyclophosphamide + doxorubicin + cisplatin	alive	118.08	118.08	pleura	unknown
**17**	cisplatin + etoposide	alive	43.53	43.53	/	/
**18**	cisplatin + etoposide	alive	31.57	31.57	/	/
**19**	/	alive	60.19	111.11	mediastinum、bone	R + multiple TKIs
**20**	cisplatin + etoposide	alive	0.36	0.36	/	/
**21**	cisplatin + etoposide	alive	78.72	168.28	mediastinum, pleura	R
**22**	paclitaxel + carboplatin	die	1.54	15.47	mediastinum、liver	C
**23**	non-platinum regimen	die	1.54	17.74	mediastinum、lung	/

**24**	octreotide	alive	60.06	60.06	/	/
**25**	cyclophosphamide + doxorubicin + paclitaxel	die	29.50	31.51	bone	C
**26**	docetaxel + cisplatin	die	9.17	23.95	lung、liver	C + intervention therapy

Abbreviation: S:surgery, R： radiotherapy, C： chemotherapy. Chemotherapy regimens refers only to the initial chemotherapy regimen. TKI: tyrosine kinase inhibitor.NA: not available. OS: overall survival, PFS: progression free survival. SLN: supraclavicular lymph node.

Figure S1. The overall survival curve of low versus intermediate/high grade TNET.

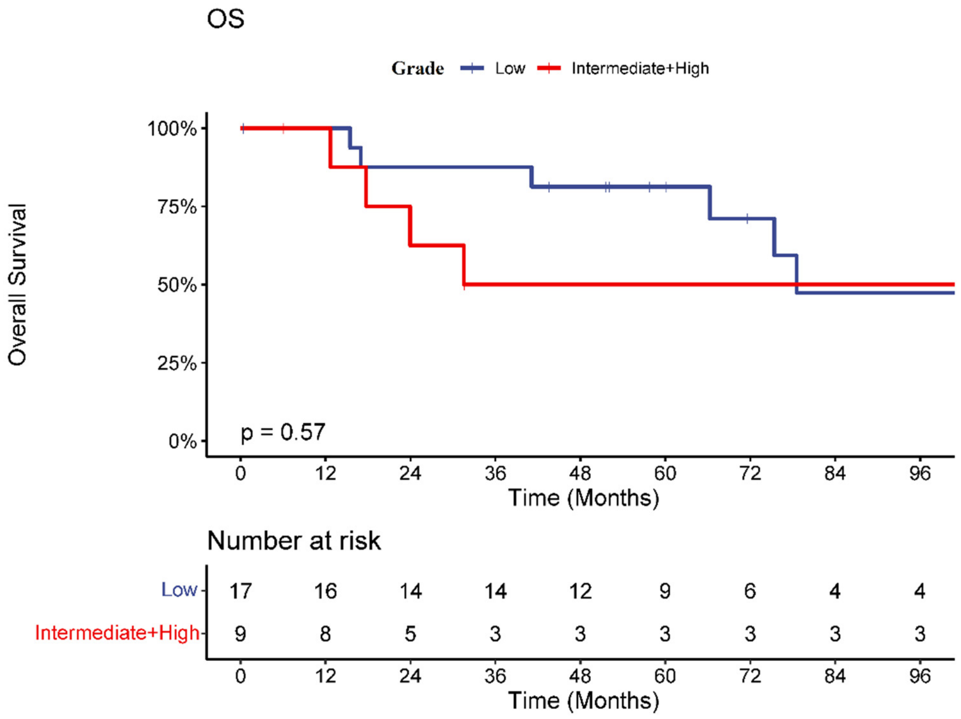

Supplement: Supplementary file 1 [file cancers-14-04944-s001.zip › cancers-1890972-supplementary.pdf]
